# Anti-inflammatory and acetylcholinesterase activity of extract, fractions and five compounds isolated from the leaves and twigs of *Artemisia annua* growing in Cameroon

**DOI:** 10.1186/s40064-016-3199-9

**Published:** 2016-09-09

**Authors:** Rosine D. K. Chougouo, Yves M. M. Nguekeu, Jean P. Dzoyem, Maurice D. Awouafack, Jonas Kouamouo, Pierre Tane, Lyndy J. McGaw, Jacobus N. Eloff

**Affiliations:** 1 Laboratory of Natural Products Chemistry, Department of Chemistry, Faculty of Science, University of Dschang, P.O. Box 67, Dschang, Cameroon; 2 Faculty of Pharmaceutical Sciences, Université des Montagnes, Bangangté, Cameroon; 3 Phytomedicine Programme, Department of Paraclinical Sciences, Faculty of Veterinary Science, University of Pretoria, Private Bag X04, Onderstepoort 0110, Pretoria, South Africa; 4 Department of Biochemistry, Faculty of Science, University of Dschang, P.O. Box 67, Dschang, Cameroon

**Keywords:** *Artemisia annua*, Phytochemical constituents, Nitric oxide, Acetylcholinesterase

## Abstract

**Background:**

Natural products, including those derived from higher plants have, over the years, contributed greatly to the development of modern therapeutic drugs. Due to the medicinal importance in traditional practice and the diversified biology and chemistry of the constituents from *Artemisia* spp., the genus has been receiving growing attention. The aim of this study was to investigate the ability of the ethanol extract, four fractions (F1–F4) and five compounds namely artemisinin (1), scopoletin (2), chrysosplenetin (3), eupatin (4) and 3-*O*-β-d-glucopyranoside of sitosterol (5) isolated from *A. annua* to modulate the activity of anticholinesterase (AchE) and the production of nitric oxide (NO) in LPS-activated RAW 264.7 macrophages.

**Results:**

At the lowest concentration tested (6.25 µg/mL), the crude extract and fraction F2 had the highest NO inhibitory activity (72.39 and 71.00 % inhibition respectively) without significant toxicity on the viability of macrophage cells (93.86 and 79.87 % of cell viability respectively). The crude extract inhibited AchE activity by 71.83 % (at 1 mg/mL) with an IC_50_ value of 87.43 µg/mL while F2 and F4 were the most active fractions (IC_50_ values of 36.75 and 28.82 µg/mL). Artemisinin (**1**) and chrysosplenetin (**3**) had the highest AChE activity with 71.67 and 80.00 % inhibition (at 0.1 mg/mL) and IC_50_ values of 29.34 and 27.14 µg/mL, respectively.

**Conclusion:**

Our results validate the traditional use of *A. annua* and could help to support the usefulness of this plant in the treatment of inflammatory and neurological disorders especially where nitric oxide and a cholinesterase are involved.

## Background

*Artemisia* L. is a genus of small herbs and shrubs found in northern temperate regions. It belongs to the important family Compositae (Asteraceae), one of the largest plant families with about 1000 genera and more than 20,000 species. Within this family, *Artemisia* is included in the tribe Anthemideae and comprises over 500 species, which are mainly found in Asia, Europe and North America (Bora and Sharma 2011). *Artemisia annua* L., commonly known as sweet wormwood or Qinghao, is a large vigorous weedy annual shrub often reaching more than 2 m tall with aromatic leaves (Wright 2002)’ The leaves produce essential oils used in folk and modern medicine, and in the cosmetics and pharmaceutical industry (Teixeira da Silva 2004). It has traditionally been used in China for the treatment of fever and chills (Ferreira and Janick 1997). Some *Artemisia* species including *A. annua* have been traditionally used in pain, inflammation and febrile conditions (Habib and Waheed 2013; Huang et al. 1993). Though originally growing in Asia and Europe, the plant is cultivated in Africa and used as a tea for the treatment of malaria (Klayman 1993). The aerial parts of *A. annua* are source of artemisinin that has potent antimalarial activity (Bhakuni et al. 2001). Besides antimalarial activity, *A. annua* has biological activities such as antioxidant, antibacterial, antifungal, anti-inflammatory, angiotensin-converting enzyme inhibitory, cytokinin-like and antitumor effects (Bhakuni et al. 2001; Chougouo-Kengne et al. 2013; Chu et al. 2014; Woerdenbag et al. 1993; Kim et al. 2015). These activities relate to the presence of secondary metabolites such as sesquiterpenoids (Chu et al. 2014), flavonoids, terpenoids, steroids, aliphatic hydrocarbons, aromatic ketones, aromatic acids, phenylpropanoids (Brown 2010), alkaloids and coumarins (Bhakuni et al. 2001).

Natural products, including those derived from higher plants have, over the years contributed greatly to the development of modern therapeutic drugs. For instance, galantamine is an anticholinesterase (AchE) inhibitor drug used for the treatment of Alzheimer’s disease, originally isolated from several plants including *Galanthus nivalus,* bulbs and flowers of *Galanthus caucasicus*, *Galanthus woronowii* and related genera like *Narcissus*, *Leucojum* and *Lycoris* including *Lycoris radiata* (Olin and Schneider 2002).

Most plant-derived secondary metabolites are known to interfere directly or indirectly with various inflammatory mediators including nitric oxide (NO). The NO radical is known to play a central role in inflammatory and immune reactions. However, excessive production of NO may cause tissue damage. It is well known that NO plays an important role in the pathogenesis of inflammatory diseases (Calixto et al. 2003). However, inflammatory processes are involved in the onset and maintenance of many severe disorders including neurodegenerative diseases such as Alzheimer’s disease (AD) (Scrivo et al. 2011). AD occurs as a result of decreased cholinergic transmission, increased oxidative stress and increased inflammatory condition (Perry et al. 1998). Due to the medicinal importance in traditional practice and the diversified biology and chemistry of the constituents from *Artemisia* spp., this genus has been receiving growing attention. The aim of this study was to investigate the ability of the ethanol extract, fractions (F1–F4) and purified compounds from *A. annua* to modulate the activity of AChE and the production of NO in LPS-activated RAW 264.7 macrophages.

## Results

**Fig. 1:**
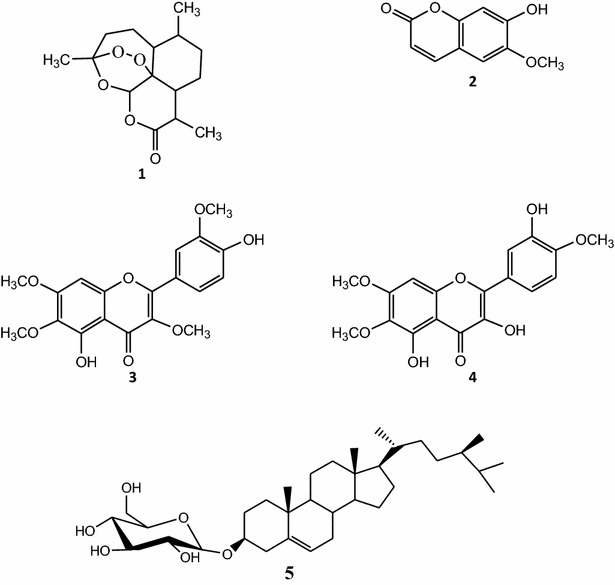
Chemical structures of compounds isolated from *Artemisia annua:* artemisinin (**1**), scopoletin (**2**), chrysosplenetin (**3**), eupatin (**4**) and 3-*O*-*β*-d-glucopyranoside of sitosterol (**5**)

The ethanol crude extract of leaves and twigs of *A. annua* subjected to repeated silica gel column chromatography and Sephadex LH-20 yielded five compounds: artemisinin (**1**) (Rimada et al. 2009), scopoletin (**2**) (Darmawan et al. (2012), chrysosplenetin (**3**) (Calcagno-pissarelli et al. 2010), eupatin (**4**) (Jiang–Jiang et al. 2010) and 3-*O*-*β*-d-glucopyranoside of sitosterol (**5**) (Al-Oqail et al. (2012) (Fig. 1). The structures of these compounds were identified by analysis of their spectroscopic data and by comparison with those reported in the literature.

Compounds were assayed for their potential to inhibit the NO production in macrophage cells. For each sample, four concentrations were used 50, 25, 12.5 and 6.25 µg/mL for the crude extract and fractions (F1–F4) then 20, 5, 2 and 0.5 µg/mL for compounds and quercetin. All the samples tested reduced NO production to some extent. The percentage of NO inhibition calculated from the amount of NO produced revealed that, at the highest concentration, the crude extract, F2 and F3 as well as compounds **3** and **4** had the highest inhibitory activity with more than 100 % inhibition relatively to the control, with the respective cell viability values of 3.94, 33.39, 2.48, 15.89 and 7.49 %. At the same high concentrations, fraction F_4_, artemisinin (**1**) and scopoletin (**2**) inhibited the NO production 95.53, 99.15 and 81.30 %, respectively without obvious cytotoxic effect (Table 1). At the lowest concentration, scopoletin (**2**) still had significant NO inhibitory activity (65.48 % inhibition).

**Table 1:** NO inhibitory activity of extract, fractions (F1–F4) and five compounds isolated from the leaves and twigs of *Artemisia annua* in LPS-activated RAW 264.7 macrophages

Samples	Concentration (µg/mL)	NO (µM)	% NO inhibition	% Cell viability
Crude extract	50	−0.32 ± 0.08	104.16 ± 1.08	3.94 ± 0.17
	25	0.05 ± 0.11	99.33 ± 1.37	41.58 ± 6.75
	12.5	0.07 ± 0.16	99.15 ± 2.09	56.41 ± 2.31
	6.25	2.12 ± 0.38	72.39 ± 4.94	93.86 ± 8.02
Fractions				
F1	50	−0.21 ± 0.09	102.78 ± 1.20	33.39 ± 5.37
	25	−0.05 ± 0.16	100.71 ± 2.09	69.94 ± 5.45
	12.5	0.38 ± 0.14	95.01 ± 1.82	77.61 ± 1.84
	6.25	3.13 ± 0.92	59.26 ± 12.00	112.41 ± 3.13
F2	50	−0.03 ± 0.16	100.36 ± 2.07	2.48 ± 0.05
	25	−0.23 ± 0.00	102.95 ± 0.00	26.49 ± 4.58
	12.5	1.87 ± 2.13	75.67 ± 7.69	54.11 ± 1.00
	6.25	2.23 ± 0.52	71.00 ± 6.74	79.87 ± 1.65
F3	50	1.43 ± 2.56	81.37 ± 3.35	2.33 ± 0.28
	25	1.69 ± 1.83	78.09 ± 3.85	69.84 ± 1.04
	12.5	2.55 ± 1.92	66.86 ± 4.91	85.78 ± 5.61
	6.25	4.79 ± 0.20	37.67 ± 2.59	103.81 ± 9.82
F4	50	0.34 ± 0.16	95.53 ± 2.09	64.17 ± 2.20
	25	1.86 ± 0.42	75.84 ± 5.49	91.14 ± 6.57
	12.5	3.64 ± 0.93	52.70 ± 2.12	102.24 ± 7.67
	6.25	6.72 ± 2.12	12.63 ± 7.53	108.48 ± 0.57
Compounds				
**1**	20	0.07 ± 0.17	99.15 ± 2.16	80.91 ± 1.71
	5	0.84 ± 0.54	89.14 ± 7.05	91.32 ± 2.20
	2	1.30 ± 0.75	83.09 ± 9.72	104.46 ± 9.66
	0.5	3.36 ± 1.10	56.33 ± 14.37	109.66 ± 6.22
**2**	20	0.12 ± 0.06	98.46 ± 0.79	81.61 ± 4.35
	5	1.71 ± 0.16	77.74 ± 2.09	88.48 ± 1.19
	2	2.15 ± 0.32	72.04 ± 4.22	69.92 ± 9.23
	0.5	2.65 ± 0.34	65.48 ± 4.41	80.34 ± 8.56
**3**	20	−0.32 ± 0.02	104.16 ± 0.30	15.89 ± 2.37
	5	0.10 ± 0.13	98.64 ± 1.67	28.49 ± 2.18
	2	1.41 ± 0.08	81.71 ± 1.04	67.39 ± 3.74
	0.5	4.79 ± 0.22	37.67 ± 2.88	95.64 ± 4.88
**4**	20	−0.20 ± 0.02	102.61 ± 0.30	7.49 ± 0.62
	5	0.17 ± 0.48	97.77 ± 6.28	17.56 ± 1.66
	2	0.16 ± 0.08	97.94 ± 1.08	48.48 ± 3.88
	0.5	1.27 ± 0.15	83.44 ± 1.96	79.34 ± 5.34
**5**	20	2.34 ± 0.30	69.62 ± 3.92	72.70 ± 1.85
	5	2.85 ± 0.38	62.89 ± 4.98	84.46 ± 6.10
	2	3.09 ± 0.50	59.78 ± 6.56	99.58 ± 9.56
	0.5	6.01 ± 0.12	21.79 ± 1.58	102.14 ± 1.66
Quercetin	20	0.20 ± 0.08	102.61 ± 1.08	23.08 ± 5.37
	5	1.10 ± 0.18	85.68 ± 2.34	76.22 ± 2.06
	2	4.38 ± 0.30	43.03 ± 3.89	98.32 ± 5.17
	0.5	5.01 ± 1.67	34.91 ± 2.76	104.92 ± 6.13

All the samples had AChE inhibitory activity, all had lower activity than galantamine. The crude extract showed 71.83 % inhibition with IC_50_ value of 87.43 µg/mL. Fractions F_2_ and F_4_ were the most actives (IC_50_ value of 36.75 and 28.82 µg/mL) (Table 2).

**Table 2:** Acetylcholinesterase inhibitory activity of extract, fractions (F1–F4) and five compounds isolated from the leaves and twigs of *Artemisia annua*

Samples	% Inhibition	IC_50_ (µg/mL)
Crude extract	71.83 ± 7.32^a^	87.43 ± 4.04^a^
Fractions		
F1	58.33 ± 6.00^b^	379.82 ± 20.16^b^
F2	88.67 ± 5.28^c^	36.75 ± 3.59^c^
F3	69.00 ± 9.26b	104.08 ± 8.60^d^
F4	91.17 ± 7.59^c,d^	28.82 ± 5.15^e^
Compounds		
**1**	71.67 ± 7.90^a,b^	29.34 ± 4.92^e^
**2**	56.34 ± 4.92^b^	70 ± 5.97^f^
**3**	80.00 ± 9.67^b,c^	27.14 ± 5.16^e^
**4**	58.22 ± 2.73^b^	67.07 ± 7.32 ^g^
**5**	68.00 ± 5.51^b^	37.73 ± 2.45^c,h^
Galantamine	88.22 ± 2.73^c^	8.22 ± 2.73^i^

Data represent the mean ± SD of three independent experiments; values with different letters are significantly different at p < 0.05, according to the Fisher’s least significant difference (LSD) analysis

## Discussion

Natural products continue to provide new and important leads for different pharmacological targets including inflammatory conditions. Therefore, in this study the extract, fractions and compounds were tested for their capacity to inhibit the production of NO in murine RAW 264.7 macrophages. Therefore, considering the high cytotoxicity (Table 1) The NO inhibition is more likely related to the cytotoxicity of the extracts and compounds on macrophages cells. However at the lowest concentration, the crude extract and fraction F_2_ remained active (72.39 and 71.00 % inhibition, respectively) without significant inhibition of the viability of macrophage cells (93.86 and 79.87 % of cell viability, respectively). These results are in accordance with the previous reported potential of extract from *Artemisia* species as inhibitors of nitric oxide production. Extracts of several species of *Artemisia* including *A. stolonifera*, *A. selengensis*, *A. japonica*, *A. montana*, *A. capillaris*, *A. sylvatica*, *A. keiskeana* and *A. scoparia* were significantly reduced the NO production at higher concentrations in the presence of LPS compared with that in control cells (Choi et al. 2013). The NO inhibitory effects observed in our study may therefore be related to the presence of artemisinin (**1**) and scopoletin (**2**) in the extract of *A. annua*. Artemisinin (**1**) and scopoletin (**2**) have been reported to exert anti-inflammatory activities (Zhu et al. 2013; Kang et al. 1999). Thus, these compounds could help to support the usefulness of the plant in the treatment of inflammatory diseases and to validate the traditional indication for this species.

The ethanol extract, fractions and the five compounds obtained from *A. annua* were tested for their in vitro anticholinesterase (AChE) activity using galantamine as a positive control. To determine the inhibition percentage, samples were tested in a preliminary screening at a single concentration of 500 µg/mL for extract and fractions, 100 µg/mL for compounds and galantamine. The activity of samples with > 50 % inhibition was further tested at different concentrations to determine the IC_50_ values. Our findings of AChE activity corroborate with several previous studies. A variety of plant species have AChE activity and so may be relevant to the treatment of neurodegenerative disorders such as AD (Amoo et al. 2012; Mukherjee et al. 2007). The most active compounds were chrysosplenetin (**3**) and artemisinin (**1**) with 80.00 and 71.67 % inhibition and IC_50_ values of 27.14 and 29.34 µg/mL respectively. Many other authors have reported the AChE activity of flavonoids and sesquiterpenes (Ji and Zhang 2006; Ibrahim et al. 2013).

## Conclusion

The current study presents evidence that the ethanol extract, fractions and compounds obtained from *A. annua* have AChE inhibitory activity and are able to prevent the production of NO. Artemisinin (**1**) and scopoletin (**2**) appeared to be responsible for the anti-inflammatory activity while chrysosplenetin (**3**) caused the antiAChE activity. The activities reported here validate the traditional use of this plant against inflammatory conditions and supports the use of *A. annua* in the treatment of inflammatory and neurological disorders where a cholinesterase and nitric oxide are involved.

## Methods

### General experimental procedures

Column chromatography was performed on silica gel Merck 60 F_254_ [(0.2–0.5 mm)] 70–230 and 230–400 mesh (Darmstadt, Germany). Pre-coated silica gel 60 F_254_ thin layer chromatography (TLC) plates (Merck; Germany) were used for monitoring fractions and spots were detected with UV light (254 and 365 nm) and then sprayed with 20 % sulphuric acid (H_2_SO_4_) or vanillin-H_2_SO_4_ reagent followed by heating to 100 °C.

### Plant Materials

The aerial parts (leaves and twigs) of *Artemisia annua* were collected before the flowering period from the scholar plantation of the Notre Dame Catholic Primary School at Bangangte in the grassfield region of Cameroon in November 2013 between 9 a.m. and 3 p.m. The sample was authenticated by a botanist of the National Herbarium of Cameroon in Yaounde where our sample was compared to the deposited specimen having a voucher number 65642 HNC/Cam.

#### Extraction and isolation

Dried leaves and twigs of *A. annua* were ground to a fine powder (3 kg) which was macerated three times with 95 % ethanol (EtOH) (24, 48 and 72 h each time) at room temperature. After filtration and removal of the solvent using rotary evaporatoration, 114.9 g of crude extract were obtained.

Part of this extract (48 g) was subjected to silica gel column chromatography (CC) eluting with hexane (Hex), Hex/Ethyl Acetate (EtOAc), EtOAc–MeOH, and MeOH in increasing polarity to afford 37 fractions of 400 mL each. After comparative TLC, four combined fractions were finally obtained as follow: **F**_**1**_ [Hex–EtOAc (100:0 and 90: 10); 17.2 g], **F**_**2**_ [Hex–EtOAc (85:15, 75:25, 65:35, 55:45 and 45:55); 15.0 g], **F**_**3**_ [Hex–EtOAc (35:65 and 0:100), EtOAc–MeOH (95:5, 90:10 and 80:20); 8.3 g], **F**_**4**_ [EtOAc–MeOH (70:30, 50:50, 30:70 and 0: 100); 5.6 g]. Part of F_2_ (14 g) was subjected to a purification silica gel CC using a gradient mixture of Hex-acetone to afford four sub-fractions coded SF_1_, SF_2_, SF_3_ and SF_4_ after comparative TLC. Sub-fraction SF_1_ was subjected to Sephadex LH-20 CC using CH_2_Cl_2_–MeOH (4:1) to afford **1** (46 mg). Sub-fraction SF_2_ was subjected to silica gel CC eluted with CH_2_Cl_2_– acetone (49:1) to yield **2** (13 mg) and a mixture of two compounds that was further separated as **3** (20 mg) and **4** (8 mg) using repeated Sephadex LH-20 fractionation.

##### *Artemisinin (****1****)*

White crystal; m.p. 155–156 °C; molecular formula: C_15_H_22_O_5_; ^1^H NMR (300 MHz) in CDCl_3_*δ*_H_: 1.31 (m, H-1), 2.00/1.44 (m, H-2a/H-2b), 2.43/2.06 (ddd, H-3a/H-3b), 5.86 (s, H-5), 1.75 (m, H-7), 1.85/1.05 (m, H-8a/H-8b), 1.75/1.05 (m, H-9a/H-9b), 3.39 (dq, H–H), 1.18 (d, 7.0 Hz, H-13), 0.98 (d, 6.0 Hz, H-14) and 1.42 (s, H-15); ^13^CNMR (75 MHz) in CDCl_3_, *δ*_C_: 172.1 (C-1), 105.4 (C-2), 93.7 (C-3), 79.5 (C-4), 50.1 (C-5), 44.9 (C-6), 37.5 (C-7), 35.9 (C-8), 33.6 (C-9), 32.9 (C-10), 25.2 (C-11), 24.8 (C-12), 23.4 (C-13), 19.8 (C-14), 12.5 (C-15).

##### *Scopoletin (****2****)*

White powder, m.p. 203–205 °C, molecular formula: C_10_H_8_O_4_; ^1^H NMR (300 MHz) in CDCl_3_, *δ*_H_: 6.20 (d, 9.4 Hz, H-3) 7.51 (d, 9.4 Hz, H-4), 6.86 (s, H-5), 6.82 (s, H -8) and 3.82 (s, OCH_3_).

##### *Chrysosplenetin (****3****)*

Yellow powder, m.p. 157–158 °C; molecular formula: C_19_H_18_O_8_; ^1^H NMR (300 MHz) in CDCl_3_, *δ*_H_: 6.50 (s, H-1) 7.66 (d, 2,1 Hz, H-2′), 7.05 (d, 8.6 Hz, H-5′), 7.71 (dd, 8.6 Hz, 2,1 Hz, H -6′), 12.61 (s, 5-OH), 5.74 (s, 4′-OH), 3. 86 (s, 3′-OCH_3_), 3.93 (s, 6-OCH_3_), 3.99 (s, 7-OCH_3_), 3.96 (s, 3′-OCH_3_); ^13^C NMR (75 MHz) in CDCl_3_*δ*_C_: 155.9 (C-2), 105.40 (C-2), 138.7 (C-3), 178.9 (C-4), 152.8 (C-5), 132.3 (C-6), 158.7 (C-7), 90.3 (C-8), 152.3 (C-9), 106.6 (C-10), 122.4 (C-1′), 110.9 (C-2′), 146.3 (C-3′), 148.4 (C-4′), 114.6 (C-5′), 122.6 (C-6′), 60.1 (3-OCH_3_), 60.9 (6-OCH_3_), 56.1 (7-OCH_3_), 56.3 (3′-CH_3_).

##### *Eupatin (****4****)*

Yellow crystal, m.p. 244–245 °C, molecular formula: C_18_H_16_O_8_; ^1^H NMR (300 MHz) in CDCl_3_*δ*_H_: 12.75 (s, 5-OH), 7.74 (dd, 3.0 Hz, 12 Hz, H-6′), 7.61 (d, 3.0 Hz, H-2′), 7.01 (d, 12 Hz, H-5′), 6.81 (s, H-8), 4.00 (CH_3_O-6), 3.88 (CH_3_O-7), 3.81 (4′-OCH_3_); ^13^C NMR (75 MHz) in CDCl_3_*δ*_*C*_: 148.2 (C-4′), 144.9 (C-3′), 121.2 (C-6′), 115.5 (C-2′), 115.3 (C-1′), 90.8 (C-8), 59.6 (6-OCH_3_), 59.3 (7-OCH_3_), 55.9 (4′-OCH_3_).

##### *β*-*Sitosterol*-*3*-*O*-*β*-*d*-*glucopyranoside (****5****)*

White powder, m.p. 272–274 °C; molecular formula: C_35_H_60_O_6_; *m*/*z* 583.

### Anti-inflammatory activity

#### Nitric oxide inhibitory activity and viability of LPS-activated RAW 264.7 macrophages

##### Cell culture

The RAW 264.7 macrophages cell line was purchased from the American Type Culture Collection (ATCC TIB-71, Rockville, MD, USA) and cultured in a plastic culture flask in DMEM containing l-glutamine supplemented with 10 % FCS and 1 % PSF solution at 37 °C with 5 % CO_2_. Cells were seeded (10^4^ per well) in 96 well-microtitre plate and activated LPS alone (control) or with samples at different concentrations. Quercetin was used as a positive control (Mu et al. 2001).

##### Measurement of NO produced

The amount of nitric oxide released was determined by the Griess reagent as reported previously (Dzoyem et al. 2015).

##### Cell viability

The number of viable cells was determined as previously described by Mosmann (1983) on the macrophage cells with few modifications. Briefly, the cells were topped up with 200 µL DMEM after removal of media. 30 µL of 15 mg/mL MTT were added to each well and cells were incubated at 37 °C with 5 % CO_2_. The medium was aspirated after 2 h, and the formazan salt formed was dissolved using DMSO. The absorbance was read at 570 nm on a BioTek Synergy microplate reader. Cell viability percentage was then calculated with reference to the control (cells with LPS only considered as 100 % viable).

### Acetylcholinesterase inhibition activity

Inhibition of acetylcholinesterase activity was determined using Ellman’s colorimetric method as previously described (Dzoyem and Eloff 2015) with a modification that galantamine (at 20 µg/mL) was used as positive control.

### Statistical analysis

Experiments were performed three times and values expressed as mean ± standard deviation. Statistical analysis was performed with GraphPad InStat Software. The Fisher’s least significant difference (LSD) at 5 % significance level was used to assess the differences between values for significance. GraphPad Prism 7 Software was used for IC_50_ calculation.

## Abbreviations

AChE: acetylcholinesterase
NO: nitric oxide
LPS: lipopolysaccharides
